# Smoking as a Permissive Factor of Periodontal Disease in Psoriasis

**DOI:** 10.1371/journal.pone.0092333

**Published:** 2014-03-20

**Authors:** Márk Antal, Gábor Braunitzer, Nikos Mattheos, Rolland Gyulai, Katalin Nagy

**Affiliations:** 1 University of Szeged, Faculty of Dentistry, Department of Operative and Esthetic Dentistry, Szeged, Hungary; 2 University of Szeged, Faculty of Dentistry, Department of Oral Surgery, Szeged, Hungary; 3 The University of Hong Kong, Faculty of Dentistry, Department of Oral Rehabilitation, Hong Kong, SAR PR China; 4 University of Szeged, Faculty of Medicine, Department of Dermatology and Allergology, Szeged, Hungary and University of Pécs, Department of Dermatology, Venerology and Oncodermatology, Pécs, Hungary; National Cancer Center, Japan

## Abstract

**Background:**

Population-based studies have identified smoking as a pathogenetic factor in chronic periodontitis. At the same time, chronic periodontal disease has also been found to occur more often in persons suffering from psoriasis than in controls with no psoriasis. It is known that smoking aggravates both periodontal disease and psoriasis, but so far it has not been investigated how smoking influences the occurrence and severity of periodontal disease in psoriasis.

**Methods:**

A hospital-based study was conducted to investigate this question. The study population consisted of 82 psoriasis patients and 89 controls. All patients received a full-mouth periodontal examination, and a published classification based on bleeding on probing, clinical attachment level and probing depth was utilized for staging. Both patients and controls were divided into smoker and non-smoker groups, and the resulting groups were compared in terms of periodontal status. Beyond the descriptive statistics, odds ratios were computed.

**Results:**

Psoriasis in itself increased the likelihood of severe periodontal disease to 4.373 (OR, as compared to non-smoker controls, p<0.05), while smoking increased it to 24.278 (OR, as compared to non-smoker controls, p<0.001) in the studied population. In other words, the risk of severe periodontal disease in psoriasis turned out to be six times higher in smokers than in non-smokers.

**Conclusions:**

The results of this study corroborate those of other studies regarding the link between psoriasis and periodontal disease, but they also seem to reveal a powerful detrimental effect of smoking on the periodontal health of psoriasis patients, whereby the authors propose that smoking may have a permissive effect on the development of severe periodontal disease in psoriasis.

## Introduction

Several chronic illnesses can be traced back to alcohol consumption, sedentary lifestyle, unhealthy nutrition or smoking. Smoking, in particular, is a global public health problem [1]. Population-based epidemiological studies have found that periodontal disease is more common in smokers than in nonsmokers [2], [3]. Beyond the obvious explanation that tobacco smoke is a local irritant, it has been shown that smoking favors colonization by specific periodontopathic bacteria [4], modulates cellular immunity [5]–[7] and favors the development and aggravation of autoimmune and immune-mediated conditions [8], [9], including psoriasis [10]. Smoking, therefore, appears to be a risk factor for both periodontal disease and psoriasis. What is more interesting is that psoriasis and periodontal disease seem to be associated themselves. It was Yamada and colleagues in 1992 who first reported a case of psoriasis in which exacerbation of the cutaneous disease was accompanied by gingival epithelial changes and periodontal bursts [11], but the first blinded, case-controlled study on the assumed association was published only eighteen years later by Preus and colleagues [12]. This was the first study to seriously raise the issue of the possible association between the two conditions, followed by the studies of Lazaridou et al. [13] and Keller and Lin [14], both of which corroborated the findings of the Preus group.

It is established that the immunopathogenesis of psoriasis [15] and the aggravation of periodontal disease [16] is linked to altered T- lymphocyte - mediated immunity. Periodontal disease turns progressive when B-cell/plasma cell responses become dominant in the periodontal inflammatory process, which is preceded by the oligoclonal expansion of Th_2_ lymphocytes. These lymphocytes stimulate B-cells and induce humoral immune responses by secreting interleukins IL-4, IL-5 and IL-10 (see Table 1). It is to be noted that Th_1_ cells are also capable of stimulating B cells by secreting INF-γ. This can be an alternative way of shifting the immune response toward B cell dominance, especially in the context of suppressed T cell responses (as it happens in the oligoclonal expansion of Th_2_ cells). Th_1_ cells are stimulated by IL-6, IL-8, IL-12, IL-18, TNF-α and INF-γ, the levels of all of which were found to be elevated in the serum of patients with chronic plaque psoriasis [17]. It might not be too far-fetched to hypothesize that psoriasis can aggravate periodontal disease because enhanced Th_1_ INF-γ secretion furthers the effects of Th_2_ dominance. However, the elaborate examination of the connection between periodontal disease and psoriasis is no subject of this study.

**Table 1:** The applied clinical staging and the corresponding pathological/pathophysiological changes.

CLINICAL STAGING	PATHOLOGY/PATHOPHYSIOLOGY
(Fernandes et al., 2009)	(Ohlrich et al., 2009)
**1.NO CLINICAL SIGNS**- no clinical attachment loss (CAL) or bleeding on probing (BOP)	(NO LESION- NOT CLASSIFIED EXPLICITLY IN OHLRICH ET AL.)
(GINGIVITIS-NOT CLASSIFIED EXPLICITLY IN FERNANDES ET AL.)(CPITN 1)	**1. INITIAL LESION** – up to 4 days following plaque accumulation. Polymorphonuclear leukocytes (PMN), complement activation, loss of connective tissue. Mast cells release tumor necrosis factor alpha, PMNs migrate into the gingival sulcus, but as the bacteria are protected by the biofilm, abortive phagocytosis occurs. PMNs release lysosomal contents, which leads to further tissue destruction.
**2.EARLY PERIODONTITIS**- CAL≥1 mm in ≥2 teeth(CPITN 2)	**2.EARLY/STABLE LESION**- 7-21 days after plaque accumulation, clinically evident approximately from day 12. Dominantly macrophages and lymphocytes (CD4^+^:CD8^+^ 2:1). Perivascular inflammatory infiltrate. Intercellular spaces between epithelial cells widen, bacterial products infiltrate the gingival tissues at a higher rate. Escalation of response. If plaque removed, tissue remodeling can take place.
**3.MODERATE PERIODONTITIS**- 3 sites with CAL ≥4 mm and at least 2 sites with probing depth (PD) ≥3 mm(CPITN 3)	**3. ESTABLISHED OR PROGRESSIVE LESION**- dominantly a B cell/plasma cell response. High levels of IL-1 and IL-6: connective tissue loss, breakdown of bone.
**4. SEVERE PERIODONTITIS**- CAL ≥6 mm in ≥2 teeth and PD ≥5 mm in ≥1 site(CPITN 4)	**4. ADVANCED LESION**- Overt loss of attachment. High levels of IL-1, TNF α and PGE_2_ stimulate fibroblasts and macrophages to produce matrix metalloproteases. The junctional epithelium progresses in apical direction (deepening periodontal pocket). Oligoclonal Th_2_ (CD4^+^) dominance.

The approximate CPITN stage is also given (in brackets).

As tobacco smoking also interferes with immunity, favors colonization by periodontopathic bacteria and acts as a local irritant, we hypothesized that smoking may act as a trigger or permissive factor of periodontal disease in patients suffering from psoriasis. To test this hypothesis, the prevalence and severity of periodontal disease was assessed in a group smoking and non-smoking psoriasis patients and a group of smoking and non-smoking psoriasis-free controls. The specific question we sought an answer to was whether periodontal disease occurs more often and in a more serious form in smoking psoriasis patients than in non-smoking psoriasis patients. The primary aim of the study, therefore, was to find evidence for or against the hypothesized permissive role of smoking.

## Methods

### Ethics statement

The study was approved by the Human Ethics Review Board of the University of Szeged (approval Nos. 2848 and 2879), and the study design conformed to the Declaration of Helsinki in all respects. Written informed consent was obtained from all participants.

### Protocol and Study Participants

A hospital-based case-control study was conducted in 2012. Participants (n = 82) were selected from the patients of the Psoriasis Outpatient Unit of the Department of Dermatology and Allergology, University of Szeged. The control group (n = 89) was recruited from people attending mandatory lung screening in the same city and the same period, on a voluntary basis. Controls were age-matched to patients, but the sex ratios were also almost identical (see Table 2.). Required sample size was calculated with G*Power 3.1.5. (University of Kiel, Germany), a software designed especially for statistical power and sample size computation [18]. The software allows the computation of achieved statistical power (post-hoc) and required sample size (a priori). As mostly categorical variables were to be analyzed, a priori sample size estimation was performed for crosstabs/chi square/contingency tables, with the following input parameters: effect size (w): 0.5; α: 0.05;power (1-β): 0.85; DF: 3. Required sample size turned out to be n = 50, but much more cases were available, whereby, also expecting dropout, it was decided that 100 cases and 100 controls would be examined. This way, even with the dropout of 18 cases and 11 controls, a statistical power over 0.9 was achieved.

**Table 2:** Descriptive statistics of the study and control groups with the characteristics of the subsamples.

	PSORIASIS		CONTROL
**Age (mean±SD, years)**	50.9±13.4		50.3±13.7
**smoker subsample**	48.5±14.0		43.3±12.4
**nonsmoker subsample**	54.7±12.9		52.9±14.7
**Sex ratio F:M (n(%):n(%))**	45(55%):37(45%)		44(49%):45(51%)
**smoker subsample**	24(60%):21(40%)		12(50%):12(50%)
**nonsmoker subsample**	23(49%):24(51%)		33(51%):32(49%)
**Smoking (n(%))**	35(43%)		24(27%)
**Periodontal status in smokers (n)**	**healthy: -**	reference category	**healthy: 10**
	**early:** 5	p<0.001	**early:** 8
	**moderate:** 13	p<0.001	**moderate:** 4
	**severe:** 17	n.s.	**severe:** 2
**Periodontal status in**	**healthy:** 10	reference category	**healthy:** 25
**nonsmokers (n)**	**early:** 13	n.s.	**early:** 19
	**moderate**: 18	p = 0.05	**moderate:** 17
	**severe:** 6	p<0.05	**severe:** 4

To keep the table transparent, percentages have been rounded to whole numbers. Note that no periodontally healthy smoker psoriasis patients were found. A trend analysis (χ^2^ with adjusted residuals) revealed a significant trend (χ^2^  = 48.074, p = 0.000), with non-smoker controls being the less likely to have any stage of periodontal disease and smoker patients being the most likely to have the severe stage. Significance values refer to the comparison of odds with healthy as the reference category (see also Table 3.).

Patients were eligible for the study if they were diagnosed with psoriasis (defined as ICD-10 L40.0-L40.9) by a dermatologist. The diagnosis of psoriasis was set up earlier than the beginning of this study in all cases, whereby dermatological examination was not regarded as a direct part of this study, neither was it listed on the informed consent forms as such. Exclusion criteria for both groups were determined based on the literature of the subject and included obesity (BMI≥30), excessive alcohol consumption, drug abuse, diabetes mellitus, oestrogen deficiency, diseases causing neutropenia and local or systemic inflammatory conditions (other than psoriasis) [19], [20]. Dropout resulted partially from meeting one or more of the exclusion criteria (especially excessive self-reported alcohol consumption), but there were subjects who quit by their own will before the intraoral examinatiom, and a number of subjects failed to provide tobacco use data.

Demographic and tobacco use data were collected by a questionnaire. Medical information on both controls and psoriasis patients was extracted from patient files and hospital records. Participants were divided into smoker and non-smoker groups, based on their self-reported current tobacco use. To minimize desirable response tendency in this crucial aspect, smoking was not pointed out to the participants as the target variable until all study procedures had been finished.

The clinical staging of periodontal disease is still a matter of debate, even though the progression of the disease is well established in pathological terms [16]. The most widely used classification is the Community Periodontal Index of the WHO (CPITN) [21]. Applicable as it may be for clinical purposes, CPITN defines rather wide categories, which carries the risk of oversampling. Therefore we decided to use the more detailed (and thus more strict) staging proposed by Fernandes and colleagues [22]. The staging requires the following parameters to be recorded: the number of missing teeth (excluding third molars), Plaque Index (PI; also known as the Silness-Löe Index), bleeding on probing (BOP; the presence or absence of bleeding within 15 sec after probing), probing depth (PD; in millimeters), and clinical attachment level (CAL; to describe the position of the soft tissue in relation to the cemento-enamel junction ).

### Clinical Examination

In order to record the above mentioned parameters, all patients received a full mouth periodontal examination, performed by a periodontist. BOP, PD and CAL were examined six sites per tooth (mesio-buccal, buccal, disto-buccal, disto-lingual, lingual, mesio-lingual), with the exception of third molars. Williams probes (Hu-Friedy Manufacturing Co., Chicago, USA) were used. Table 1 shows the categories of the applied staging and the corresponding pathological/pathophysiological status.

### Statistical Analysis

The study and control groups were compared by smoking status. To express the probability that a member of a given group develops a given clinical degree of periodontal disease, multinomial logistic regression was conducted and the odds ratios were calculated. In the multinomial model, disease severity (healthy, early, moderate, severe) was defined as the outcome variable, smoking status (smoker or non-smoker), and group (psoriasis or control) were defined as factors, and smoking intensity (expressed as the number of cigarettes smoked per day) was defined as a covariate. Descriptive statistics and odds ratios were calculated in SPSS 17.0 (SPSS, Inc., Chicago, USA).

## Results

### The population

Table 2. shows the descriptive statistics of both the study and the control groups. Females were slightly over-represented in the psoriasis group (n_female_ = 45,n_male_ = 37) while in the control group the sexes were represented almost perfectly equally (n_female_ = 44, n_male_ = 45). Crosstab analysis with χ^2^ test was conducted to find out if sex was significantly associated with disease severity, but the association turned out to be non-significant for both patients (χ^2^  = 5.184 p =  0.189) and controls (χ^2^ = 3.029, p = 0.387), whereby sex was not included in the multinomial regression. Patients and controls were age-matched (mean age for the entire sample: 50.6 years). The division into four groups by diagnosis (psoriasis or control) and smoking status (yes or no) for the purposes of the analysis did not change these parameters significantly, that is, the ratios remained representative of the whole sample. Group membership (i.e. patient or control) was shown to be significantly associated with disease severity (χ^2^ =  27.337, p = 0.000).

**Figure 1:**
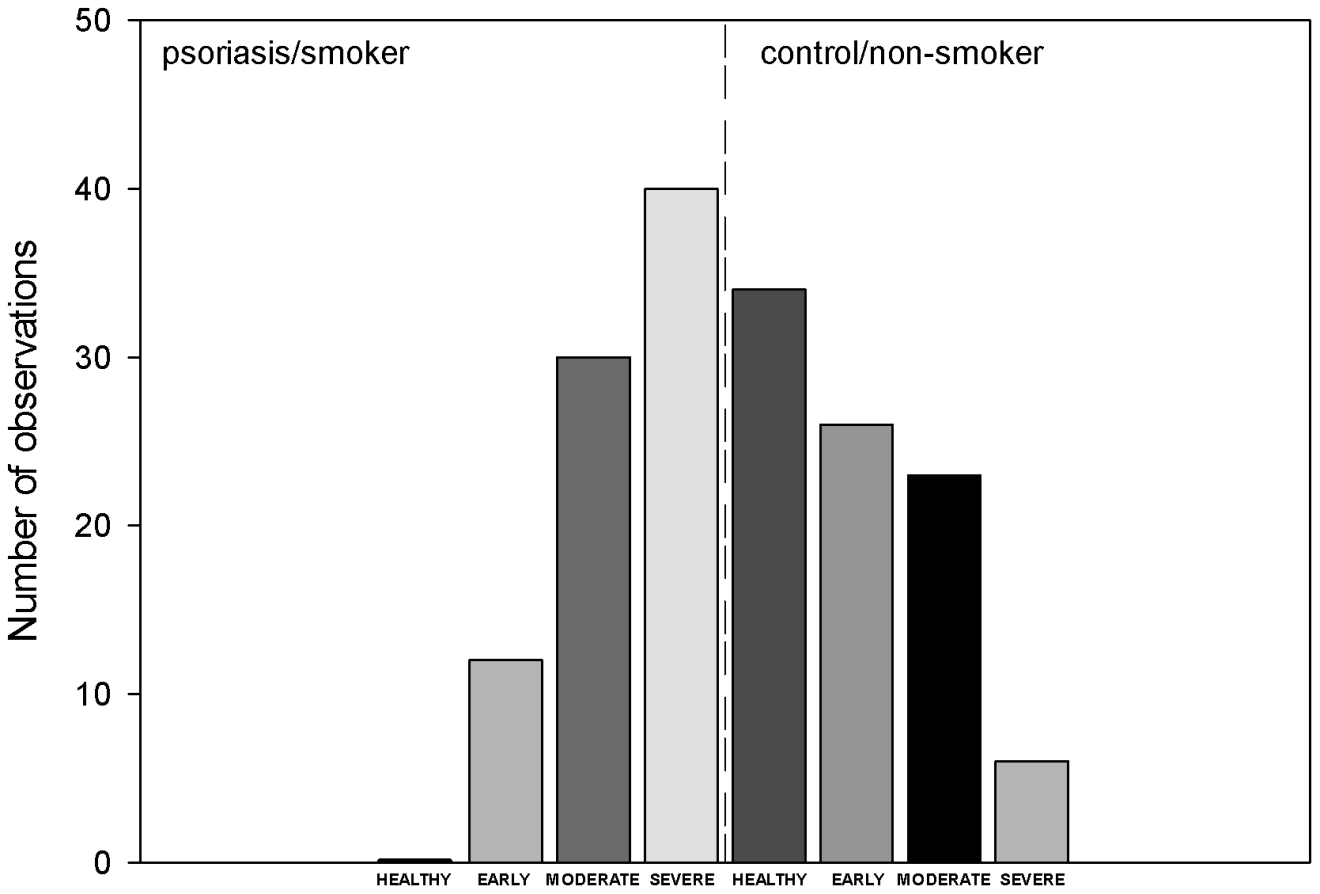
Opposing tendencies: while in the smoker patient subsample the severe stage of periodontal disease was the most frequent, and no periodontally healthy patient was seen, in the non-smoker control subsample exactly the opposite was observed.

In the psoriasis group 35 persons defined themselves as smoker (43%). The number of smokers was lower among the control group, only 24 persons defined themselves as smoker (27%). In the psoriasis+smoker group no patient could be categorized as periodontally healthy. 14% of this group had early, 37% moderate and 49% severe periodontal disease. Among non-smoker patients 21% were classified as healthy, 28% as having early, 39% as having moderate and 12% as having severe periodontal disease. 42% of smoking controls was periodontally healthy, 33% had early, 17% moderate and 8% severe periodontal disease. Nonsmoking controls were periodontally healthy in 38%, 29% had early periodontal disease, while in 26% of the cases the disease presented in its moderate and in 7% in its severe form. These data reflect opposing tendencies between smoker patients and non-smoker controls, that is, while in the smoker patient subsample the severe stage of periodontal disease is the most frequent, and no periodontally healthy patient is seen, in the non-smoker control subsample exactly the opposite can be observed (Figure 1.).

### Smoking history and smoking intensity

In terms of smoking history (expressed in years) and their intensity of smoking (expressed as the number of cigarettes smoked per day), controls and psoriasis patients turned out to be quite similar. Smoking controls had smoked for a mean of 20.9 years (mode: 20 years, SD: 11.97), and they smoked a mean of 16 cigarettes a day (mode: 20 cigarettes, SD: 11.67). Smoking patients had smoked for an average of 16.57 years (mode: 20 years, SD: 12.37), and they smoked an average of 13 cigarettes a day (mode: 20 cigarettes, SD: 8.57). Indeed, the two groups exhibited no statistically significant difference in either of these parameters (MWU = 315, n1 = 35, n2 = 24, p = 0.17, years; MWU = 758, n1 = 35, n2 = 24, p = 0.57, cigarettes). The crosstab analysis revealed that smoking status in itself was significantly associated with disease severity only in patients (χ^2^ =  18.258, p = 0.000). In controls, no significant association was seen (χ^2^ =  0.935, p = 0.812). However, when the data were re-coded so that the outcome variable was not disease severity, but the occurrence of any stage of the disease, the association was highly significant (χ^2^ =  89.000, p = 0.000). As for smoking history and intensity, the built-in likelihood ratio test of the multinomial logistic regression module of SPSS revealed that smoking history had a significant effect on disease severity (χ^2^ = 11.099, p = 0.011), but the effect of smoking intensity was not significant (χ^2^ =  30.862, p = 0.504).

### Odds Ratios

Odds ratios with significance and confidence intervals are given in Table 3. Odds ratios were calculated in a multinomial logistic regression model. Smokers and controls were compared stagewise, according to the applied classification. The factors of the model were smoking status (smoker or non-smoker) and group (patient or control), and the covariate was smoking history (in years). Five comparisons were done, that is, non-smoker controls were compared with smoker controls (OR_early_: 1.053, OR_moderate_: 0.588, OR_severe_: 1.250 ), with non-smoker patients (OR_early_: 1.944, OR_moderate_: 2.647, OR_severe_: 4.373 ) and with smoker patients (OR_early_: 1.711, OR_moderate_: 2.500, OR_severe_: 24.278), smoker patients were also compared with non-smoker patients (OR_early_: 6.082, OR_moderate_: 1.712, OR_severe_: 4.480 ), and smoker controls were compared with smoker patients (OR_early_: 1.904, OR_moderate_: 9.900, OR_severe_: 2.589 ). Significantly higher odds of developing moderate (p = 0.05) and severe (p<0.05) periodontal disease were found when non-smoker patients were compared against non-smoker controls (the periodontal risk of psoriasis alone). The highest odds ratio resulted from the comparison of smoker patients with non-smoker controls (24.278, p<0.001), which is 4.43 times higher than the combination of the individual odds of smoking (1.250, n.s.) and psoriasis (4.373, p<0.05). It must be noted, though, that the high odds ratio had a rather wide confidence interval. When compared with non-smoker patients, smoker patients’ odds of developing periodontal disease was significantly higher at all three stages (p<0.001). Finally, as compared to smoker controls, smoker patients’ odds of developing early or moderate periodontal disease were also significantly higher (p<0.001), but no significant difference was observed regarding the severe stage of the disease.

**Table 3:** Odds ratios by clinical staging.

COMPARISON	STAGE	OR	Sig.	95% CI (lower;upper)
**SMOKER CONTROLS (vs. non-smoker controls)**	**early**	1.053	n.s.	0.394;3.177
	**moderate**	0.588	n.s	0.158;2.187
	**severe**	1.250	n.s	0.197;7.942
**NON-SMOKER PATIENTS (vs. non-smoker controls)**	**early**	1.944	n.s.	0.706;5.353
	**moderate**	2.647	*p = 0.05*	0.985;7.113
	**severe**	4.373	*p<0.05*	1.114;17.169
**SMOKER PATIENTS (vs. non-smoker controls)**	**early**	1.711	n.s	0.618;4.732
	**moderate**	2.500	n.s	0.924;6.761
	**severe**	24.278	*p<0.001*	5.207;113.189
**SMOKER PATIENTS (vs. non-smoker patients)**	**early**	6.082	*p<0.001*	1.516;2.440
	**moderate**	1.722	*p<0.001*	3.535;3.689
	**severe**	4.480	*p<0.001*	1.651-6.851
**SMOKER PATIENTS (vs. smoker controls)**	**early**	1.904	*p<0.001*	1.202; 3.016
	**moderate**	9.900	*p<0.001*	1.568;6.262
	**severe**	2.589	n.s.	2.108;2.695

In each case, the ratios express the odds that a member of the given category (printed in capitals) develops the given stage of periodontitis in comparison with the members of another category (in brackets). Significance levels and 95% confidence intervals (with lower and upper limits) are also given.

The covariate smoking history was significantly associated with disease severity in both patients (χ^2^ = 12.204, p = 0.014) and controls (χ^2^ = 9.869, p = 0.02). The odds ratios were positive for the moderate and severe stages in both groups (OR_control, moderate_: 1.145, CI:0.999–1.312; OR_control, severe_: 1.164, CI: 0.963–1.408; OR_patient, moderate_: 1,112, CI: 0.939–1.318; OR_patient, severe_:1.185, CI: 0.998–1.406), indicating that a longer smoking history significantly increases the odds that the moderate or severe stage of the disease occurs, as compared to the early stage.

## Discussion

In the present study it was demonstrated that psoriasis patients who smoke are at an approximately sixfold higher risk of developing severe periodontal disease, as compared to psoriasis patients who do not smoke. Psoriasis in itself increases the likelihood of severe periodontal disease to 4.373 (as compared to non-smoker controls), while smoking appears to increase this to 24.278. In other words, the risk of severe periodontal disease in psoriasis is approximately six times higher in smokers than in non-smokers. It would be reasonable to infer that this increase in risk is merely the result of the individual risk factors adding up. To test that possibility, odds ratios were combined and the synergy factor was calculated.

Combined odds expresses the probability of a given condition if all combined risk factors are present. For samples of similar size, combination of odds is done by averaging their logs (*logOR_1_/logOR_2_*). For psoriasis alone (4.373) and smoking alone (1.250), the combined odds of severe periodontal disease is 6.60, far below the observed 24.278. From this it may be conferred that what is observed in smoker patients is not simply the odds of psoriasis and smoking added up. Computation of the synergy factor corroborates this suspicion. The synergy factor was devised by Borja and co-workers [23] to allow the estimation of whether two factors are in a positive or negative interaction in terms of bringing about a given condition, based on their individual odds. The factor is calculated as *SF*  = *OR*_12_/(*OR*_1_ × *OR*_2_), where *OR*_12_ expresses the combined odds of the examined factors. SF = 1 expresses the simple addition of the odds, SF<1 means negative interaction (antagonism) and SF>1 denotes positive interaction (synergy). The value of the synergy factor for the odds of psoriasis and smoking is 1.2, indicating a synergistic effect. It seems, therefore, that smoking acts as a permissive factor.

It was also found that the association between smoking status and disease severity is significant only in the patient group, which is also to support that exposure to cigarette smoke and psoriasis act together toward the aggravation of periodontal disease. The lack of significant association, however, should not be interpreted as evidence that cigarette smoke alone does not cause periodontal disease. Evidence suggests that it does [24], and the additional analysis also showed that when the target variable is the occurrence of periodontal disease, the association is highly significant. From this it might be inferred, that smoking increases the odds of periodontal disease in both controls and psoriasis patients, but psoriasis patients are perdisposed to the more severe forms of the disease.

A further interesting result is the significant association of smoking history with the occurrence of the moderate and severe forms of periodontal disease in both groups. Together with the lack of significant association with the number of cigarettes smoked per day, it suggests that the key factor in the proposed permissive role of cigarette smoke is chronic exposure, apparently regardless of how heavy a smoker one is.

As no study before has dealt with the effects of smoking on periodontal disease severity in psoriasis, it is beyond our limitations to give an exact explanation of these findings. However, enough is known about the pathogenesis of psoriasis and periodontal disease and the pathophysiological effects of smoking to allow the articulation of the elements of a hypothesis, which we briefly delineate here.

As pointed out by Ohlrich and co-workers, the most readily observable difference between the early and later stages of periodontal disease is that in the later stages the inflammatory response is dominated by B lymphocytes, instead of T lymphocytes [16]. It is in the context of this exaggerated and maladaptive B cell/plasma cell response that periodontal dissue destruction and bone breakdown occurs. Our propsed hypothesis, therefore, centers around this T-to-B shift, which appears to be the key event in the aggravation of periodontal disease, and how cigarette smoke might play a permissive role in the process. Here we concentrate only on a putative immunological backrgound, while we understand that additional factors, like the detrimental effect of smoking on gingival circulation [25], [26] can also contribute to the end result.

It is well established that psoriasis is associated with a millieu of inflammatory mediators that favors a shift toward B cell responses. Serum levels of the inflammatory mediators IL-6, IL-8, IL-12, IL-18, TNF-α and INF-γ were found to be elevated in plaque psoriasis [17]. These mediators are all documented to activate B cells [27]–[30]. TNF-α is also a potent stimulant of fibroblasts and macrophages, which, upon activation, release matrix metalloproteases [16]. As mentioned before, periodontal disease itself is associated with Th_2_ expansion. This, on one hand, suppresses T cell responses, but Th_2_ cells, in the presence of antigen, can stimulate B cells too [31]. Therefore, it might be argued that psoriasis increases susecptibility to destructive immune responses in general by shifting the immune response profile toward B cell responses, which would explain the original observation - also corroborated by this study- that patients with psoriasis develop periodontal disease more often than psoriasis-free individuals.

How, then, might cigarette smoke boost this effect? We propose that toll-like receptor 4 (TLR 4) can play a key role here. Toll-like receptors are preferentially expressed on immune cells, and mediate immune responses. TLR4 on the surface of Th1 cells activate these cells upon binding to bacterial lipopolysaccharides to secrete INF-γ, a potent B cell activator [32]. Normally, this is part of the immune response to bacterial colonization of the gingival sulcus. However, if TLR 4 is overexpressed, the response may become exaggerated and maladaptive. Pace and co-workers demonstrated that cigarette smoke causes upregulation of TLR4 in airway epithelial cells of smokers with COPD, and they proposed that this upregulation might be the reason why a dominantly Th1-regulated immune response is seen in the airways of COPD patients [33]. The authors also demonstrated that cigarette smoke increased epithelial cells’ ability to bind bacterial lipopolysaccharides [33]. TLR4 upregulation was demonstrated in the psoriatic skin too [34]. It is not known if cigarette smoke has the same effect in the periodontal tissues (and on the surface of Th1 cells), but it seems plausible to assume that TLR 4 upregulation can be the key step in the destructive T- to B- shift in periodontal disease in the context of an already B-biased inflammatory mediator environment. Based on the literature, we propose that the examination of the effects of cigarette smoke on TLR4 expression in the gingival tissues could be a logical next step toward the understanding of our observations.

## Conclusions

The results of this study corroborate those of other studies regarding the link between psoriasis and periodontal disease, but they also seem to reveal a powerful detrimental effect of smoking on the periodontal health of psoriasis patients, whereby we propose that smoking might have a permissive effect on the development of severe periodontal disease in psoriasis. It seems plausible to propose that smoking exerts this effect through the upregulation of TLR-4, but this cannot be decided without further research, as at the moment no data are available regarding the periodontium.

## References

[1] Edwards R (2004) The problem of tobacco smoking. BMJ 328: 217–219. 14739193 10.1136/bmj.328.7433.217 PMC318495

[2] Beck JD, Koch GG, Rozier RG, Tudor GE (1990) Prevalence and risk indicators for periodontal attachment loss in a population of older community-dwelling blacks and whites. J Periodontol 61: 521–528. 2391631 10.1902/jop.1990.61.8.521

[3] Tomar SL, Asma S (2000) Smoking-attributable periodontitis in the United States: findings from NHANES III. National Health and Nutrition Examination Survey. J Periodontol 71: 743–751. 10.1902/jop.2000.71.5.743 10872955

[4] Kubota M, Tanno-Nakanishi M, Yamada S, Okuda K, Ishihara K (2011) Effect of smoking on subgingival microflora of patients with periodontitis in Japan. BMC Oral Health 11: 1. 21208407 10.1186/1472-6831-11-1 PMC3020163

[5] Korn S, Wiewrodt R, Walz YC, Becker K, Mayer E, et al (2005) Characterization of the interstitial lung and peripheral blood T cell receptor repertoire in cigarette smokers. Am J Respir Cell Mol Biol 32: 142–148. 15539458 10.1165/rcmb.2004-0239OC

[6] Sopori M (2002) Effects of cigarette smoke on the immune system. Nat Rev Immunol 2: 372–377. 12033743 10.1038/nri803

[7] Sullivan AK, Simonian PL, Falta MT, Mitchell JD, Cosgrove GP, et al (2005) Oligoclonal CD4+ T cells in the lungs of patients with severe emphysema. Am J Respir Crit Care Med 172: 590–596. 15937291 10.1164/rccm.200410-1332OC PMC2718531

[8] Masdottir B, Jonsson T, Manfredsdottir V, Vikingsson A, Brekkan A, et al (2000) Smoking, rheumatoid factor isotypes and severity of rheumatoid arthritis. Rheumatology (Oxford) 39: 1202–1205. 11085797 10.1093/rheumatology/39.11.1202

[9] Mathews JD, Whittingham S, Hooper BM, Mackay IR, Stenhouse NS (1973) Association of autoantibodies with smoking, cardiovascular morbidity, and death in the Busselton population. Lancet 2: 754–758. 4126476 10.1016/s0140-6736(73)91037-4

[10] Emre S, Metin A, Demirseren DD, Kilic S, Isikoglu S, et al.. (2012) The relationship between oxidative stress, smoking and the clinical severity of psoriasis. J Eur Acad Dermatol Venereol. 10.1111/j.1468-3083.2012.04700.x 23004342

[11] Yamada J, Amar S, Petrungaro P (1992) Psoriasis-associated periodontitis: a case report. J Periodontol 63: 854–857. 1403594 10.1902/jop.1992.63.10.854

[12] Preus HR, Khanifam P, Kolltveit K, Mork C, Gjermo P (2010) Periodontitis in psoriasis patients: a blinded, case-controlled study. Acta Odontol Scand 68: 165–170. 20141361 10.3109/00016350903583678

[13] Lazaridou E, Tsikrikoni A, Fotiadou C, Kyrmanidou E, Vakirlis E, et al (2013) Association of chronic plaque psoriasis and severe periodontitis: a hospital based case-control study. J Eur Acad Dermatol Venereol 27: 967–972. 22703187 10.1111/j.1468-3083.2012.04615.x

[14] Keller JJ, Lin HC (2012) The effects of chronic periodontitis and its treatment on the subsequent risk of psoriasis. Br J Dermatol 167: 1338–1344. 22755552 10.1111/j.1365-2133.2012.11126.x

[15] Schon MP, Boehncke WH (2005) Psoriasis. N Engl J Med 352: 1899–1912. 15872205 10.1056/NEJMra041320

[16] Ohlrich EJ, Cullinan MP, Seymour GJ (2009) The immunopathogenesis of periodontal disease. Aust Dent J 54 Suppl 1S2–10. 19737265 10.1111/j.1834-7819.2009.01139.x

[17] Arican O, Aral M, Sasmaz S, Ciragil P (2005) Serum levels of TNF-alpha, IFN-gamma, IL-6, IL-8, IL-12, IL-17, and IL-18 in patients with active psoriasis and correlation with disease severity. Mediators Inflamm 2005: 273–279. 16258194 10.1155/MI.2005.273 PMC1533889

[18] Faul F, Erdfelder E, Lang AG, Buchner A (2007) G*Power 3: a flexible statistical power analysis program for the social, behavioral, and biomedical sciences. Behav Res Methods 39: 175–191. 17695343 10.3758/bf03193146

[19] Genco RJ (1996) Current view of risk factors for periodontal diseases. J Periodontol 67: 1041–1049. 10.1902/jop.1996.67.10.1041 8910821

[20] Genco RJ, Borgnakke WS (2013) Risk factors for periodontal disease. Periodontol 2000 62: 59–94. 23574464 10.1111/j.1600-0757.2012.00457.x

[21] Barmes D (1994) CPITN—a WHO initiative. Int Dent J 44: 523–525. 7836006

[22] Fernandes JK, Wiegand RE, Salinas CF, Grossi SG, Sanders JJ, et al (2009) Periodontal disease status in gullah african americans with type 2 diabetes living in South Carolina. J Periodontol 80: 1062–1068. 19563285 10.1902/jop.2009.080486 PMC2862011

[23] Cortina-Borja M SD, Combarros O, Lehmann DJ (2009) The synergy factor: a statistic to measure interactions in complex diseases. BMC Research Notes 2. 10.1186/1756-0500-2-105 PMC2706251 19527493

[24] Tonetti MS (1998) Cigarette smoking and periodontal diseases: etiology and management of disease. Ann Periodontol 3: 88–101. 9722693 10.1902/annals.1998.3.1.88

[25] Mavropoulos A, Brodin P, Rosing CK, Aass AM, Aars H (2007) Gingival blood flow in periodontitis patients before and after periodontal surgery assessed in smokers and non-smokers. J Periodontol 78: 1774–1782. 17760548 10.1902/jop.2007.060472

[26] Morozumi T, Kubota T, Sato T, Okuda K, Yoshie H (2004) Smoking cessation increases gingival blood flow and gingival crevicular fluid. J Clin Periodontol 31: 267–272. 15016254 10.1111/j.1600-051X.2004.00476.x

[27] Hirano T, Yasukawa K, Harada H, Taga T, Watanabe Y, et al (1986) Complementary DNA for a novel human interleukin (BSF-2) that induces B lymphocytes to produce immunoglobulin. Nature 324: 73–76. 3491322 10.1038/324073a0

[28] Falkoff RJ, Muraguchi A, Hong JX, Butler JL, Dinarello CA, et al (1983) The effects of interleukin 1 on human B cell activation and proliferation. J Immunol 131: 801–805. 6602846

[29] Durali D, de Goer de Herve MG, Giron-Michel J, Azzarone B, Delfraissy JF, et al (2003) In human B cells, IL-12 triggers a cascade of molecular events similar to Th1 commitment. Blood 102: 4084–4089. 12893768 10.1182/blood-2003-02-0518

[30] Rieckmann P, D'Alessandro F, Nordan RP, Fauci AS, Kehrl JH (1991) IL-6 and tumor necrosis factor-alpha. Autocrine and paracrine cytokines involved in B cell function. J Immunol 146: 3462–3468. 2026875

[31] Del Prete G (1998) The concept of type-1 and type-2 helper T cells and their cytokines in humans. Int Rev Immunol 16: 427–455. 9505198 10.3109/08830189809043004

[32] Netea MG, Van der Meer JW, Sutmuller RP, Adema GJ, Kullberg BJ (2005) From the Th1/Th2 paradigm towards a Toll-like receptor/T-helper bias. Antimicrob Agents Chemother 49: 3991–3996. 16189071 10.1128/AAC.49.10.3991-3996.2005 PMC1251502

[33] Pace E, Ferraro M, Siena L, Melis M, Montalbano AM, et al (2008) Cigarette smoke increases Toll-like receptor 4 and modifies lipopolysaccharide-mediated responses in airway epithelial cells. Immunology 124: 401–411. 18217953 10.1111/j.1365-2567.2007.02788.x PMC2440834

[34] Curry JL, Qin JZ, Bonish B, Carrick R, Bacon P, et al (2003) Innate immune-related receptors in normal and psoriatic skin. Arch Pathol Lab Med 127: 178–186. 12562231 10.5858/2003-127-178-IIRRIN

