# Correction: Smoking as a Permissive Factor of Periodontal Disease in Psoriasis

**DOI:** 10.1371/journal.pone.0110975

**Published:** 2014-10-08

**Authors:** The PLOS ONE Staff

The values in the y-axis of figure 1 are incorrect. The authors have provided a corrected version here.

**Figure 1:**
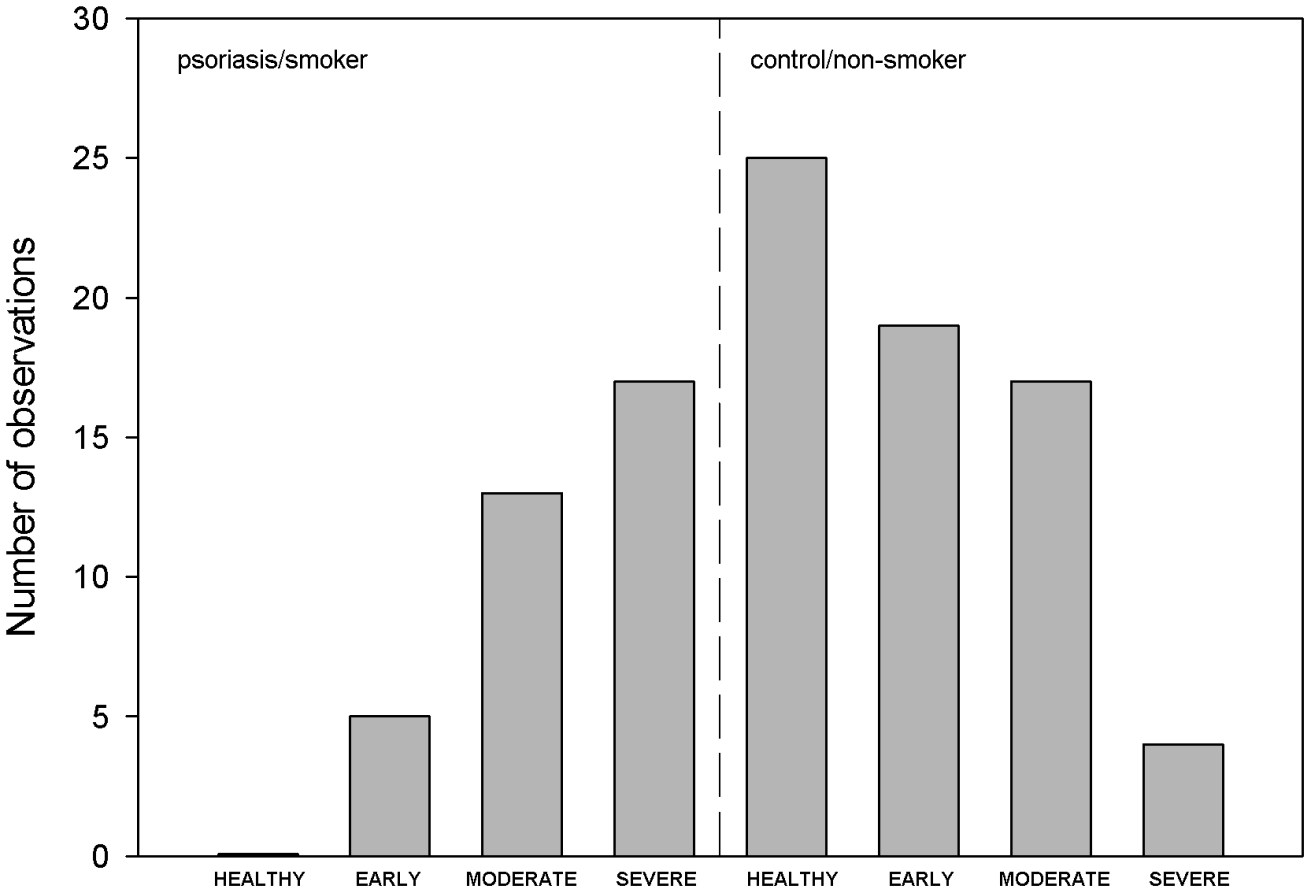
Opposing tendencies: while in the smoker patient subsample the severe stage of periodontal disease was the most frequent, and no periodontally healthy patient was seen, in the non-smoker control subsample exactly the opposite was observed.
